# A survey by the European Society of Breast Imaging on the utilisation of breast MRI in clinical practice

**DOI:** 10.1007/s00330-017-5121-4

**Published:** 2017-11-22

**Authors:** Paola Clauser, Ritse Mann, Alexandra Athanasiou, Helmut Prosch, Katja Pinker, Matthias Dietzel, Thomas H. Helbich, Michael Fuchsjäger, Julia Camps-Herrero, Francesco Sardanelli, Gabor Forrai, Pascal A. T. Baltzer

**Affiliations:** 1 Department of Biomedical Imaging and Image-guided Therapy, Division of Molecular and Gender Imaging, Medical University of Vienna/General Hospital Vienna, Waehringer Guertel 18-20, 1090 Vienna, Austria; 2 Department of Radiology, Radboud University Nijmegen Medical Centre, Geert Grooteplein Zuid 10, 6525 GA Nijmegen, The Netherlands; 3 Department of Radiology, Division of Breast Imaging, “MITERA” Hospital, 6 Erythrou Stavrou Street, 151 23 Athens, Greece; 4 Institute of Diagnostic Radiology, University Hospital Erlangen, Maximiliansplatz 1, 91054 Erlangen, Germany; 5 Division of General Radiology, Department of Radiology, Medical University of Graz, Auenbruggerplatz 9/P, 8036 Graz, Austria; 6 Department of Radiology, Hospital de la Ribera, Carretera de Corbera, Km. 1, 46600 Alzira, Valencia Spain; 7 Department of Biomedical Sciences for Health, University of Milan, Milan, Italy; 8 Department of Radiology, IRCCS (Research Hospital) Policlinico San Donato, Via Morandi 30, 20097, San Donato Milanese, Milan, Italy; 9 Department of Radiology, Duna Medical Center, Lechner Ödön fasor 7, Budapest, 1095 Hungary

**Keywords:** Breast, Magnetic resonance imaging, Survey and Questionnaires, Practice Guideline, Radiologists

## Abstract

**Objectives:**

While magnetic resonance imaging (MRI) is considered a helpful diagnostic tool in breast imaging, discussions are ongoing about appropriate protocols and indications. The European Society of Breast Imaging (EUSOBI) launched a survey to evaluate the utilisation of breast MRI in clinical practice.

**Methods:**

An online survey reviewed by the EUSOBI board and committees was distributed amongst members. The questions encompassed: training and experience; annual breast MRI and MRI-guided-intervention workload; examination protocols; indications; reporting habits and preferences. Data were summarised and subgroups compared using χ^2^ test.

**Results:**

Of 647 EUSOBI members, 177 (27.4%) answered the survey. The majority were radiologists (90.5%), half of them based in academic centres (51.9%). Common indications for MRI included cancer staging, treatment monitoring, high-risk screening and problem-solving, and differed significantly between countries (p≤0.03). Structured reporting and BI-RADS were mostly used. Breast radiologists with ≤10 years of experience preferred inclusion of additional techniques, such as T2/STIR (p=0.03) and DWI (p=0.08) in the scan protocol. MRI-guided interventions were performed by a minority of participants (35.4%).

**Conclusions:**

The utilisation of breast MRI in clinical practice is generally in line with international recommendations. There are substantial differences between countries. MRI-guided interventions and functional MRI parameters are not widely available.

***Key points*:**

*• MRI is commonly used for the detection and characterisation of breast lesions.*

*• Clinical practice standards are generally in line with current recommendations.*

*• Standardised criteria and diagnostic categories (mainly BI-RADS) are widely adopted.*

*• Younger radiologists value additional techniques, such as T2/STIR and DWI.*

*• MRI-guided breast biopsy is not widely available.*

## Introduction

Magnetic resonance imaging (MRI) is widely used for the detection and characterisation of breast lesions [1]. Due to many reasons, including the relatively high cost and its limited availability, MRI is utilised mainly for selected indications such as screening modality in high-risk women, preoperative evaluation of disease extent of specific breast cancer subtypes, assessment of response to neoadjuvant therapy [2–6]. However, indications vary due to clinical preferences, official recommendations [2, 7, 8], as well as local health care system reimbursement policies, which do change over time as the body of evidence evolves [9–12].

Another issue contributing to inter-institutional and international variations in the use of breast MRI are uncertainties regarding image acquisition and interpretation. While there is consensus about the fact that contrast-enhanced sequences are mandatory in breast MRI, the usage and value of additional techniques or specific reading protocols and criteria remain a matter of debate. Furthermore, the on-going debate about the impact of preoperative MRI regarding surgical outcomes stresses the importance of MRI-guided interventions for diagnosis and treatment planning.

In this context, the European Society of Breast Imaging (EUSOBI) decided to launch a survey among its members to gather representative data on how breast MRI is currently used in clinical practice. The results of this survey are reported in this paper.

## Materials and methods

### Survey design and distribution

Two board certified radiologists, one with more than 10 years of experience in breast imaging and breast MRI and one with a background in survey methodology, developed the survey. The questions encompassed: training and experience; annual breast MRI and MRI-guided intervention workload; indications and technical details of the MRI examination; and reporting habits and preferences. The full questionnaire is available online (https://de.surveymonkey.com/r/RRPQHNJ?sm=xQclpzxR8w3M4Q%2btaA9q2A%3d%3d). Once the survey was reviewed and approved by members of the EUSOBI executive board and committees the survey was published online, using a dedicated software platform (SurveyMonkey, Palo Alto, CA). EUSOBI members were invited to participate by an email that contained a link to the survey, which was sent out from the central EUSOBI office. The survey was available online for six weeks, and two reminders were sent out during this period, by email and on the EUSOBI Facebook page (https://www.facebook.com/eusobieuropeansociety/?ref=aymt_homepage_panel).

### Data analysis

**Fig. 1:**
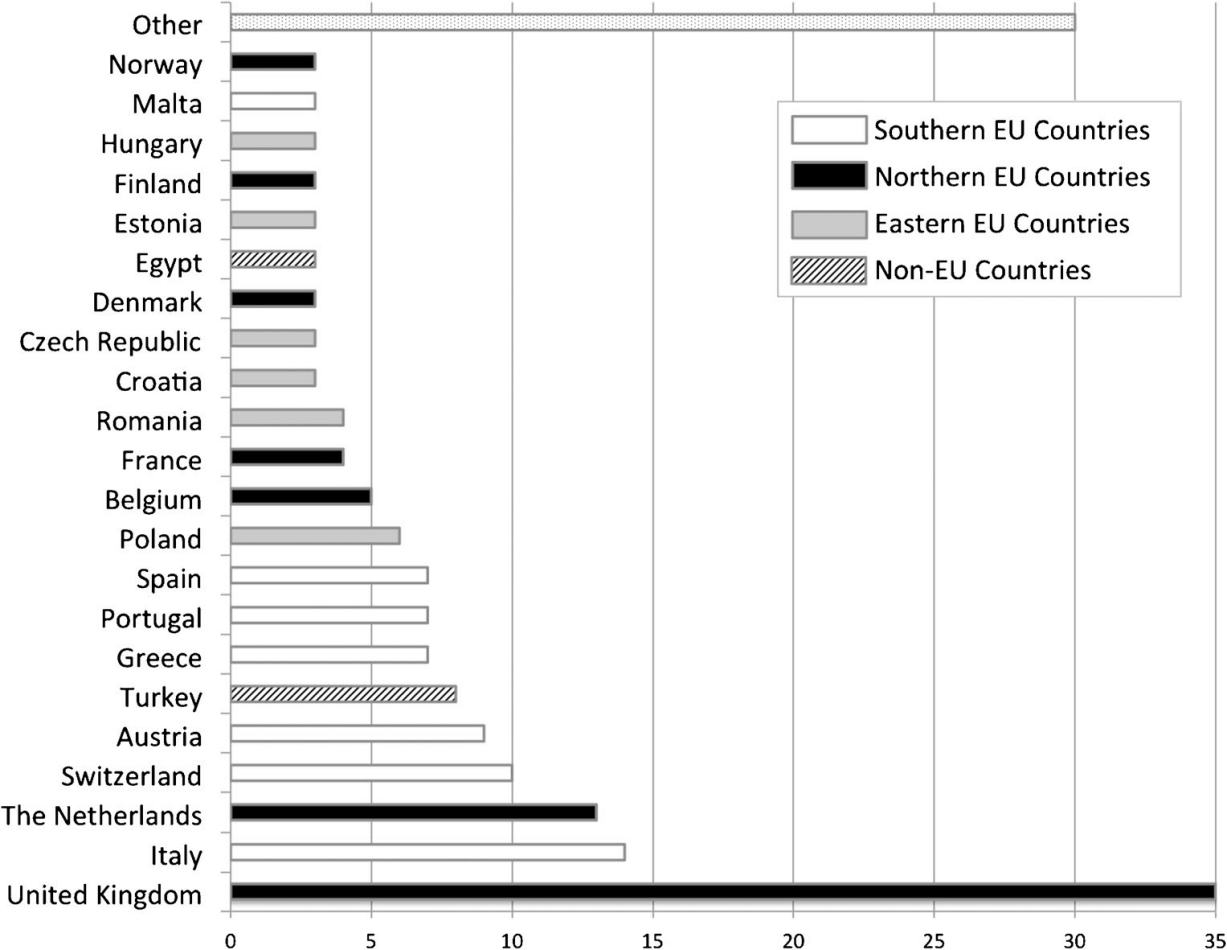
Countries where the participants were working at the time of the survey (3/189 did not answer, 1.6%). Four different geographical areas were distinguished: southern; northern and eastern European countries; and non-European countries. Southern and northern countries were considered together as western European countries. Other: countries of various geographical areas in which only one person answered the survey. Footnotes: The number of responders is indicated in the horizontal -axis

After the survey was closed, spreadsheet data were exported for statistical analysis. Responses to the questions were extracted and summarised. Based on this first data evaluation, data were separated into groups, considering different types of institutions (academic, community, private), and different geographical areas (e.g. southern Europe vs. northern Europe as defined in Figure 1). The separation in different geographical areas was obtained by evaluating clustered data. Thus, the countries of participants that returned similar answers were considered together. The responses between the subgroups were compared using the χ^2^ test. Statistical analysis was performed using SPSS v.20 for Windows (IBM, Armonk, New York, USA).

Data were reported as frequencies and percentages. Shapiro-Wilk test was used to test for normal distribution. Mean and standard deviation (SD) were used in the case of normal or near-normal distribution, median and interquartile range (IQR) in the case of non-normal distribution.

## Results

A total of 189 survey participants were noted. Of these, 177 confirmed that they were EUSOBI members, yielding a response rate among members of 27.4% (Figure 1).

**Fig. 2:**
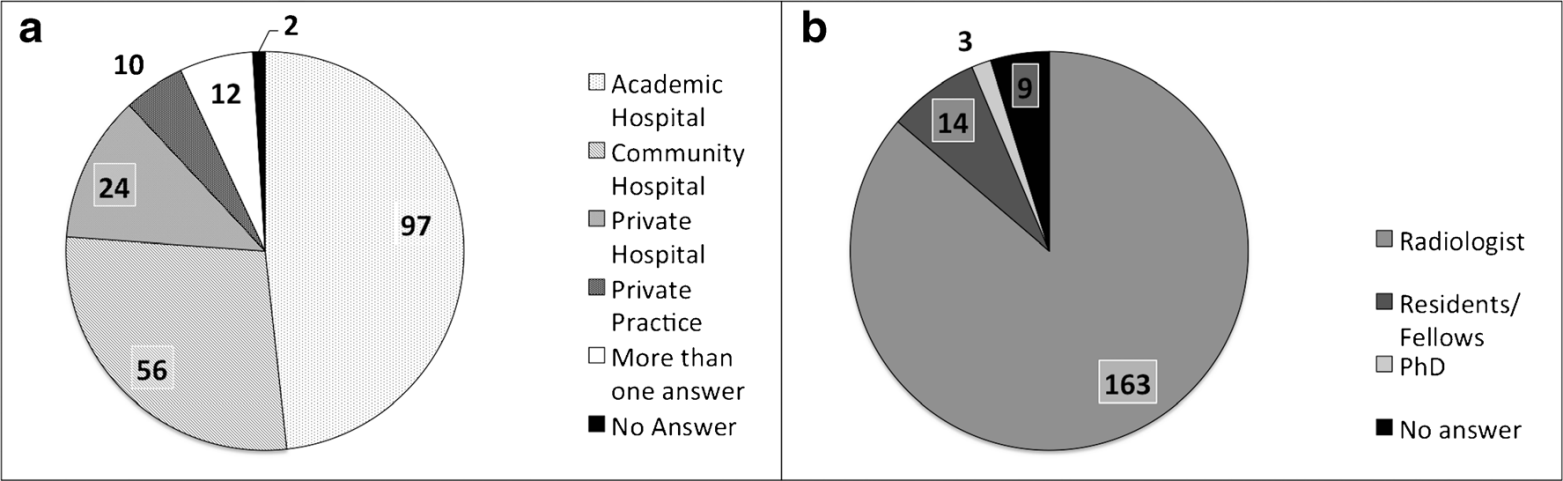
Clinical setting (a) and current position (b) of the people who participated in the survey

More than half the participants were based at an academic centre (51.9%) (Figure 2a). Board-certified radiologists, primarily, responded to the survey (90.5%) (Figure 2b). Further details are given in Table 1.

**Table 1:** Years of experience in breast imaging and breast MRI of the participants and number of examinations per year performed by the centres

	Range	Median; IQR
Experience in breast imaging	0 - 40	12; 14
Experience in breast MRI	0 - 25	6; 8
Breast MRI/year	10 - 4000	200; 300
MR-guided interventions/year	2 - 350	20; 60

IQR: Interquartile range

### Indications for breast MRI

**Fig. 3:**
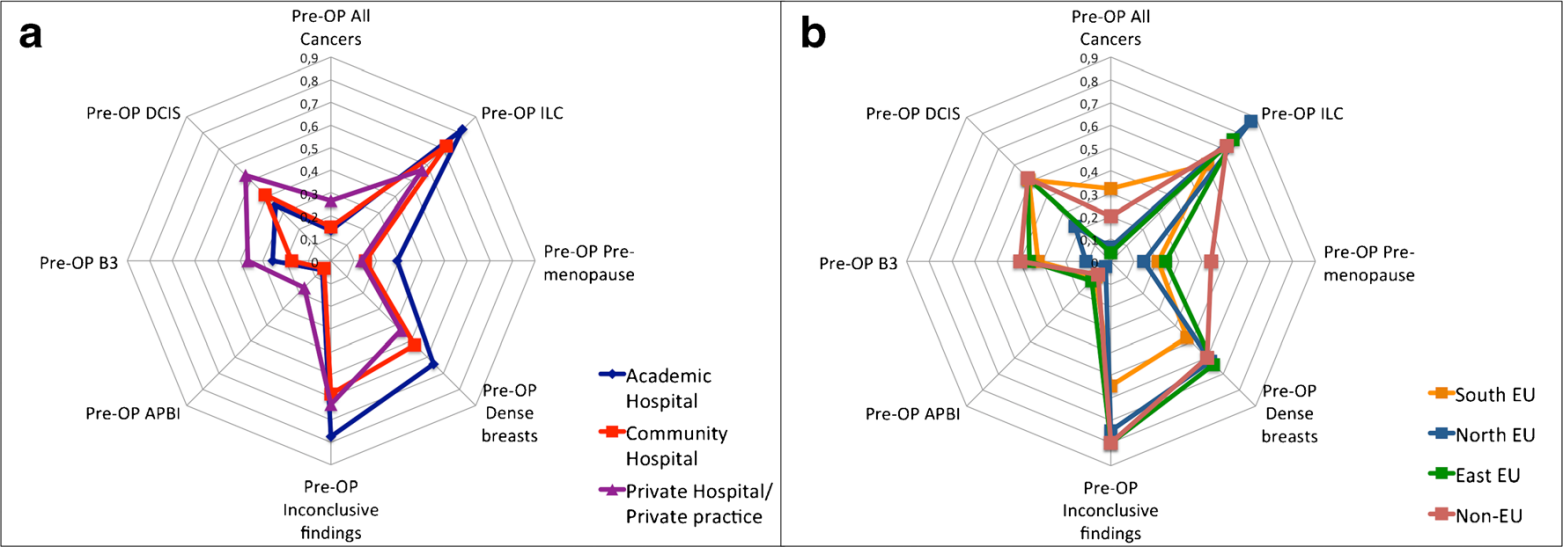
Indications for pre-operative breast MRI in different clinical settings (a) and geographical areas (b). Footnotes: Pre-OP: pre-operative MRI; ILC: invasive lobular carcinoma; APBI: accelerated partial breast irradiation; DCIS: ductal carcinoma in situ; B3: high-risk lesions/lesions with uncertain malignant potential

**Fig. 4:**
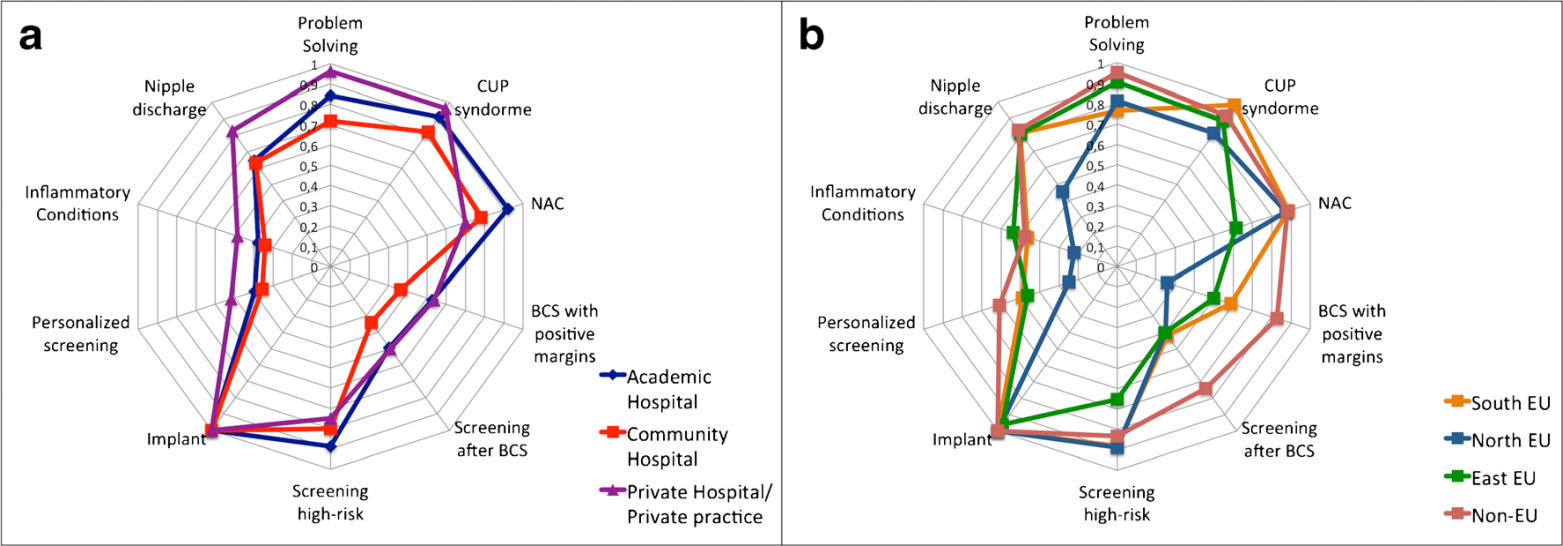
Common indications for breast MRI in the different clinical settings. Footnotes: CUP: carcinoma of unknown primary; NAC: neoadjuvant chemotherapy; BCS: breast-conserving surgery

The most common indication was preoperative MRI in women with breast cancer (Table 2, Figure 3), followed by evaluation of breast implants (Table 2, Figure 4).

**Table 2:** Indications for which breast MRI was used by the responders

Indications	Positive answers % (Yes/Total)*
Pre-operative MRI	100 (162/162)
ILC	75.9 (123/162)
Inconclusive findings	70.9 (115/162)
Dense breasts	58.0 (94/162)
DCIS	40.7 (66/162)
B3	25.9 (42/162)
Pre-menopause	22.8 (37/162)
All Cancers	15.7 (27/162)
APBI	7.4 (12/162)
Breast implants	99.4 (164/165)
CUP syndrome	89.6 (147/164)
Neoadjuvant Chemotherapy	84.2 (139/165)
Inconclusive findings	83.9 (117/141)
Screening in high-risk	83.9 (136/162)
Nipple discharge	67.3 (111/165)
BCS with positive margins	48.4 (78/161)
Screening after BCS	45.3 (73/161)
Personalised screening	40.5 (66/163)
Inflammatory conditions	38.6 (63/163)

*Percentages are calculated considering only positive (yes) or negative (no) answers. When a question was not answered or the answer was “I don’t know”, the answer was excluded from the calculation

ILC: invasive lobular cancer; DCIS: ductal carcinoma in situ; APBI: accelerated partial breast irradiation; CUP: carcinoma of unknown primary; BCS: breast conserving surgery

Evaluation of implants was mainly performed in patients with symptoms and inconclusive findings on conventional imaging (114/165, 69.1%). Screening in the absence of a clinical complaint was rarely performed (9.7%).

Monitoring of neoadjuvant therapy with breast MRI was more frequently performed in academic centres (92.1% compared to 78.3% in community hospitals and 70% in private hospitals, p=0.006). The difference between academic and non-academic centres was particularly evident when the evaluation of early response was considered, which was significantly more common in academic centres (84.3%, 67.4%, and 48.4% respectively, p=0.001).

Breast MRI, in patients with nipple discharge was performed mainly in cases with inconclusive findings on conventional imaging (101 of 166 answers, 60.8%), while only 6.6% always performed MRI in women with nipple discharge.

### Geographical areas and their influence on indications for MRI

Indications varied between geographical regions. In southern countries (as defined in Figure 1), preoperative breast MRI was more often performed in all cancer patients rather than only for invasive lobular carcinoma (32.1%, compared to 6% in northern countries, p<0.001), and in patients with ductal carcinoma in situ (50.9%, compared to 22.2% in northern countries, p=0.003), or lesions with uncertain malignant potential (32.1% compared to 11.1%, p=0.007) (Figure 3).

In northern countries, several other indications were less frequently encountered: carcinoma of unknown primary (80.9% of the cases compared to ≥ 88.5% in the other areas, p=0.030); positive margins after breast-conserving surgery (25.8% compared to ≥ 50% in the other areas, p=0.010); personalised screening (25.0% compared to ≥ 46.1%, p=0.007); inflammatory conditions (22.2% compared to ≥ 46.1%, p=0.008); and nipple discharge (45.3% compared to ≥ 80.8%, p<0.001) (Figure 4).

Screening in high-risk women and evaluation of response during neoadjuvant therapy were less frequently used in eastern countries (Figure 1) compared to western (northern and southern countries in Figure 1) and non-EU countries (65.4% compared to ≥ 83.3%, p=0.036, and 61.5% compared to 87.3%, p=0.010, respectively).

### Reporting habits, diagnostic features, and MRI protocol preferences

Morphology was considered to provide the most relevant diagnostic information (Table 3). Opinions differed significantly when considering readers’ experience. Readers with more than 10 years of experience in breast imaging found the information of enhancement curves more important than did less-experienced readers (p=0.042). However, readers with less than 10 years of experience in breast imaging and breast MRI tended to prefer multiparametric assessment and gave higher usefulness scores for STIR/T2-weighted imaging (p=0.030), DWI (p=0.080), and MRS (p=0.117) as compared with radiologists with more years of experience. The value of morphology was considered high according to both groups (p>0.566).

**Table 3:** Responders’ opinion on the impact of diagnostic criteria

Diagnostic Value	Enhancement Curves	Morphology	T2w sequences	DWI	MRS
High	57 (41.3)	125 (91.2)	46 (34.8)	36 (29.3)	5 (6.4)
Intermediate	73 (52.9)	10 (7.3)	73 (55.3)	57 (46.3)	17 (21.8)
Low	8 (5.8)	2 (1.5)	13 (9.8)	30 (24.4)	56 (71.8)
Tot answer	138 (73.0)	137 (72.5)	132 (69.8)	123 (65.1)	78 (41.3)
No answer	51 (27.0)	52 (27.5)	57 (30.2)	66 (34.9)	111 (58.7)

Percentages are given in brackets

DWI: Diffusion Weighted Imaging; MRS: magnetic resonance spectroscopy

Most participants utilised the general Picture Archiving and Communication System (PACS) viewer and/or a dedicated MRI workstation as well as a standardised setup for reporting (Table 4). No differences were found between the different settings (p>0.238), nor when considering the experience in breast imaging and breast MRI (≤ 10 years versus > 10 years, p>0.122).

**Table 4:** Details on reporting habits

	% (positive /total answers)
Reporting viewer	
PACS	40.4 (57/141)
Scanner software	2.8 (4/141)
MRI workstation	38.3 (54/141)
Multiple systems	18.4 (26/141)
Reporting setting	
Standardised	64.5 (91/141)
Flexible	35.5 (50/141)
Report style	
Free text	17.4 (24/138)
Structured reporting	8.7 (12/138)
Structured reporting and free text	73.9 (102/138)
Diagnostic criteria	
BI-RADS only	33.6 (47/140)
BI-RADS and additional features	53.6 (75/140)
Non BI-RADS	12.8 (18/140)
Rating systems	
BI-RADS only	46.4 (65/140)
Other scoring systems	4.3 (6/140)
Empirical only	12.1 (17/140)
Combination of the above	37.1 (52/140)

The American College of Radiology Breast Imaging Reporting and Data System (BI-RADS) [13] was used for description and rating of the examination in the majority of the cases, alone or in combination with further criteria or rating systems (Table 4). Structured reporting was widely used, usually combined with free-text (Table 4).

Reporting preferences did not show significant differences related to the clinical setting (p>0.137) or to experience (p>0.168).

Answers regarding the technical protocols for breast MRI are provided In Table 5. A dedicated breast coil with at least four channels, and an automated injector for contrast medium application were used by almost all responders. While the majority of responders preferred three-dimensional (3D) gradient-echo sequences for dynamic, contrast-enhanced MRI, two-dimensional (2D) gradient-echo sequences were used by almost one-fourth of the survey participants. Fat saturation was favoured over non-fat-saturated sequences for both T1- and T2-weighting (77% used fat-saturated T1-weighted sequences alone and 71.4% used fat-saturated T2-weighted sequences alone, or along with non-fat-saturated sequences). A large fraction (60%) of the survey participants did acquire DWI regularly. Only a small number of participants (2%) made regular use of MRS.

**Table 5:** Answers on technical details of the examination

Question						
	**1/1.5T**	**1.5T**	**3T**	**1.5/3T**	**Total answers**	**No answer**
Type and operating magnet field strength	1 0.6%	94 57.3%	37 22.6%	32 19.5%	164	25
Is there a dedicated breast coil (best equipment) in your institution?	**Yes**	**No**	**Don’t know**		**Total answers**	**No answer**
	158 97%	4 2%	2 1%		164	25
If yes, how many channels?	**<7**	**≥7**	**Don’t know**		**Total answers**	**No answer**
	16 18%	69 79%	3 3%		88	101
Do you use an injector for contrast medium	**Yes**	**No**	**Don’t know**		**Total answers**	**No answer**
	135 82%	18 11%	11 7%		164	25
Flow rate?	**<2 ml/s - manual**	**≥2 ml/s**	**Don’t know**		**Total answers**	**No answer**
	7 11%	45 74%	9 15%		61	128
Contrast Medium dose?	**0.1—0.15 mmol/kg**	**0.2 mmol/kg**	**Don’t know**		**Total answers**	**No answer**
	97 68%	29 20%	16 11%		142	47
Which kind of dynamic sequence do you use?	**2D**	**3D**	**Both**		**Total answers**	**No answer**
	30 21%	104 73%	8 6%		142	47
Do you prefer fat saturation in dynamic imaging?	**Yes**	**No**	**Don’t know**		**Total answers**	**No answer**
	109 77%	24 17%	8 6%		141	48
If you are using fat saturation, which kind do you prefer?	**Spectral fat-sat**	**SPAIR**	**Other**		**Total answers**	**No answer**
	41 42%	42 43%	15 15%		98	91
Which T2-weighted sequence do you use?	**TSE w/o fat-sat**	**TSE with fat-sat**	**Both**		**Total answers**	**No answer**
	46 29%	81 50%	34 21%		164	28
Orientation of T2w imaging	**Axial**	**Sagittal**	**Coronal**	**More than one**	**Total answers**	**No answer**
	126 80%	1 1%	3 2%	27 17%	157	32
Do you use Diffusion Weighted Imaging?	**Yes**	**Selected cases**	**No**		**Total answers**	**No answer**
	85 60%	21 15%	36 25%		142	47
Do you use MR spectroscopy?	**Yes**	**Selected cases**	**No**		**Total answers**	**No answer**
	3 2%	19 14%	116 84%		138	51

### Breast interventions

Only a minority of the participants (35.4%) used MR-guided interventions, either wire-localisations or needle biopsies. Of these 67 radiologists, 29 performed both biopsies and wire localisations (43.3%), three used wire localisation exclusively (4.5%), and 35 used biopsies exclusively (52.2%). The use of planning software was more common than manual planning (27 vs 17, 40.3% vs 25.4%), and sometimes both were used together (14.9%).

MRI interventions, both wire localisation and biopsy, were more often performed in an academic environment (p<0.004).

## Discussion

The utilisation of breast MRI in clinical practice is generally in line with international recommendations. There are substantial differences between countries, regarding setting and reader experience. MRI-guided interventions and functional MRI parameters are not widely available.

### Indications for breast MRI

The survey responders actual use of breast MRI in different clinical situations agrees with the current guidelines and statements from various societies [2, 3, 7]. However, substantial differences between different settings and countries were observed: in southern countries, preoperative breast MRI is less limited to specific breast cancer patient groups. This seems to reflect the indications of current American practice parameters [3], which suggest the use of MRI to define lesion extent and muscle involvement, regardless of the histology, or to screen for undetected contralateral cancers, although this subject is still under debate [6]. Differences in clinical practice are also likely related to the acceptance of preoperative MRI by other specialists on the multidisciplinary team, particularly surgeons.

The assessment of response during or after neoadjuvant therapy was more commonly performed in academic centres: one may assume that a greater number of women are treated with neoadjuvant therapy in institutions involved in clinical trials.

The evaluation of the response to treatment and screening of high-risk women are less frequent indications in eastern countries. This latter might reflect the current lack of organised high-risk screening programs but both may also be connected to cost-issues in situations where potential clinical effects are not immediately perceived [14, 15].

While problem-solving as an indication for breast MRI is still controversial according to the literature [2, 3], our results show that breast MRI is commonly performed in patients with inconclusive findings. This was true regardless of the setting and/or the geographical area. The term ‘problem-solving’ is used for largely different clinical situations, thus creating a wide heterogeneity in the available data and evidence. This might, at least partially, explain the divergence between guidelines and clinical practice [10, 16].

Further differences between southern and northern countries regarded indications such as evaluation of lesions of uncertain malignant potential (B3), nipple discharge, inflammation, personalised screening, and evaluation after breast-conserving surgery. Northern countries seem to guide their practice more strictly in line with the available evidence that is still limited regarding specific indications [17–22].

Breast MRI was only performed by a minority of survey participants to check for implant rupture in asymptomatic women. In the United States, MRI has been suggested as a screening modality for implant rupture in asymptomatic women [23], but there is currently no evidence of its positive impact on patient treatment and outcomes.

### Reporting habits, diagnostic criteria and examination protocols

The interpretation of breast MRI is a challenging task due to the abundance of diagnostic criteria. Most participants of this survey considered morphology the most important feature when evaluating a lesion, along with the signal intensity time curve type and the lesion appearance on T2-weighted sequences. We found that younger generations of breast radiologists gave more importance to multiparametric breast MRI, including T2-weighted as well as DWI. This indicates a personal choice of young generations, who prefer to report breast MRI looking at all available multiparametric data and do not focus solely on dynamic post-contrast images. Several studies already proved the usefulness of these approach [24, 25]. In the last years, many centres – in particular academic centres - introduced in their protocols both fat-suppressed and non fat-suppressed T2 as well as DWI. Thus, younger generations are more used to working with this kind of images. Whether our results are related to a paradigm shift towards multiparametric imaging or simply reflect personal preferences with the more familiar technique was not investigated. In line with published recommendations, most survey responders preferred a standardised setting for image evaluation and reporting. For the latter, the use of BI-RADS [13] was as widespread as expected. This must be considered a big advantage in the community of breast specialists: the use of well-defined descriptors and diagnostic categories provides a common language, especially important for a diagnostic tool like breast MRI that is technically complex, with interpretation based on a variety of diagnostic criteria.

### Additional techniques

Additional MRI techniques such as DWI and MRS have been investigated for more than a decade. While there is common acceptance that MRS is still impractical in the clinical setting [26, 27], several studies demonstrate that evaluation with DWI can improve breast MRI accuracy, particularly specificity [24, 25, 28]. Despite that, only slightly more than half of the survey participants regularly applied DWI, and the technique was considered not very important for image interpretation by one-quarter of the survey participants. This may change in the future, as reflected by the body of evidence given above, and the fact that radiologists with less than 10 years of experience in breast MRI considered DWI (and, to some degree, MRS as well) more helpful for lesion diagnosis, compared to their more experienced colleagues.

Of note, MRI protocols may in the future be tailored to specific clinical indications: while a comprehensive diagnostic scan e.g. in the preoperative setting may profit from a sophisticated multiparametric protocol [29, 30], a screening examination may rather be as short as possible to enable high patient throughput [31–33].

Regarding examination protocols, the vast majority of the centres follow current state-of-the-art recommendations [2, 3, 34]. Despite the traditional belief that non-fat-saturated images are more commonly used in European countries compared to the USA, we found that fat-saturated, T1-weighted sequences are most commonly used to acquire dynamic contrast-enhanced images. Fat suppression can simplify the evaluation if significant movement artefacts are present [34].

### Breast interventions

The necessity to perform MRI-guided interventions for lesions visible only on MR images is stressed by a cancer rate around 20% in these lesions [35–40]. Although centres with a sufficient caseload are recommended to provide MRI-guided biopsies, our survey shows a different reality. Most survey participants did not offer MRI-guided breast interventions.

Considering these results, the importance of targeted (or “second-look”) ultrasound must be emphasised. Targeted ultrasound is a widely available approach to avoid further MRI interventions and follow-ups in a substantial percentage of patients [38]. However, for lesions not identifiable by ultrasound, MR-guided interventions are a necessity. Without these, MRI findings cannot be translated into clinical strategies [41].

### Limitations

This survey specifically targeted EUSOBI members and is, therefore, potentially biased towards radiologists with a special interest in breast imaging. As is typical for surveys, the response rate was below 50% [14, 15]. Participants were not evenly distributed among European countries, a fact that is also attributable to the distribution of members within the society, with the best-represented countries being Italy, the Netherlands, and United Kingdom, while other countries such as Germany and France are less represented within the EUSOBI. Furthermore, national differences in the amount of screening and assessment examinations performed in outpatient settings and private hospitals as compared to academic centres may further contribute to these heterogeneities and thus explain the imbalance of responders per country. The grouping into different regions is due to clustered survey data. While the association of some countries with specific regions does not reflect the UN definition of European regions (http://unstats.un.org/unsd/methods/m49/m49regin.htm#europe), they reflect a similarity of clinical practice in breast MR application. Our survey required already more than 10-15 minutes. To achieve an acceptable compliance, it was not possible to add further questions regarding several other issues (breast MRI acceptance between clinicians, reasons for differences between countries and institutions, use of alternative methods for MR-guided interventions). A further survey could be of interest to analyse the points that were only raised within the current study.

### Conclusions

Our survey is the first to reveal data about the actual use of MRI of the breast by the members of the EUSOBI. While substantial differences regarding several subtopics between countries of residence were noted, the following can be concluded:

1. Despite on-going controversial discussions, MRI is used for breast cancer staging and problem-solving by most responders, regardless of the setting.
2. Standardised diagnostic criteria and documentation (mainly BI-RADS) are generally adopted.
3. Breast radiologists with ≤10 years of experience preferred additional techniques, such as DWI, hinting at a generation- rather than an evidence-based influence on the use of these techniques.
4. There is no “European” breast MRI technique, as protocols vary among users. The majority of responders apply fat-saturated protocols both in T1w and T2w imaging.
5. MRI-guided breast interventions are not widely available, which must be considered as a serious weakness for a more intense distribution of this technique.

The data presented allow for a better perception of the current use of breast MRI within the EUSOBI and in Europe, and highlight the need to improve the availability of MRI-guided interventions in European countries
